# A Lateral Flow Protein Microarray for Rapid and Sensitive Antibody Assays

**DOI:** 10.3390/ijms12117748

**Published:** 2011-11-09

**Authors:** Jesper Gantelius, Tarek Bass, Ronald Sjöberg, Peter Nilsson, Helene Andersson-Svahn

**Affiliations:** 1 Division of Nanobiotechnology, School of Biotechnology, KTH-Royal Institute of Technology, Albanova University Center, 106 91 Stockholm, Sweden; jesper.gantelius@biotech.kth.se; 2 Division of Molecular Biotechnology, School of Biotechnology, KTH-Royal Institute of Technology, Albanova University Center, 106 91 Stockholm, Sweden; tarek.bass@biotech.kth.se; 3 Division of Proteomics, School of Biotechnology, KTH-Royal Institute of Technology, SciLifeLab, 171 21 Solna, Sweden; ronald.sjoberg@biotech.kth.se (R.S.); peter.nilsson@biotech.kth.se (P.N.)

**Keywords:** point of care, protein microarray, lateral flow

## Abstract

Protein microarrays are useful tools for highly multiplexed determination of presence or levels of clinically relevant biomarkers in human tissues and biofluids. However, such tools have thus far been restricted to laboratory environments. Here, we present a novel 384-plexed easy to use lateral flow protein microarray device capable of sensitive (<30 ng/mL) determination of antigen-specific antibodies in ten minutes of total assay time. Results were developed with gold nanobeads and could be recorded by a cell-phone camera or table top scanner. Excellent accuracy with an area under curve (AUC of 98% was achieved in comparison with an established glass microarray assay for 26 antigen-specific antibodies. We propose that the presented framework could find use in convenient and cost-efficient quality control of antibody production, as well as in providing a platform for multiplexed affinity-based assays in low-resource or mobile settings.

## 1. Introduction

Protein microarrays are widely used tools for diagnostic biomarker analysis, with proven utility in such varied fields of human medicine as for instance autoimmunity, allergy, infection and cancer [1]. In particular, the ability of protein microarrays to simultaneously determine presence or levels of a large number of biomolecules is useful as a means to screen a clinical sample for a variety of different conditions such as cancers or autoimmune diseases that have not yet started to manifest as symptoms or other detectable signs of disease [2], or it can serve to refine the resolution in the differentiation between for instance infections from closely related bacterial hosts [3]. Further, protein microarrays may improve the diagnostic accuracy in cancer diagnosis [4]. As all the individual microspot assays simultaneously taking place on a microarray are directed to the exact same sample, the potential variability arising from aliquot preparations for the same number of simplex-assays is also conveniently avoided.

The assay procedures for protein microarrays are low- to moderately labor-intensive, and may due to the ambient-analyte conditions governing the microspot assay dynamics offer excellent sensitivity on par with ELISAs [1]. However, due to the many assay steps involved and the required convective mixing in the sample incubation step, microarray assays are typically carried out manually, requiring skilled personnel and well-equipped laboratories. Further, as fluorescent probes remain the preferred choice for detection due to excellent sensitivity and high dynamic range, expensive and bulky laser-equipped high-end scanners are usually needed.

Manual handling and advanced detection strategies result in assay times normally spanning several hours and consequently current microarray technologies are not well suited for mobile, on-site or point of care applications. While it is likely that a large proportion of microarray analyses could preferentially continue to be performed within advanced health care sites such as hospitals or centralized laboratories, portable microarray solutions may support more rapid clinical decision making in emergency situations in the field or in such areas where little healthcare infrastructure exists. Further, point of care microarray tests might serve to relieve health care resources when patients or care-givers are able to conduct regular testing themselves. Finally, such portable tests could facilitate those unable to go to a hospital or health care point for testing [5].

While there is a wide variety of simple portable tests commercially available, including for instance rapid tests for sexually transmitted diseases, cardiovascular disease and allergies, such tests are typically simplex or low-plex, with limited quantitative ability and sensitivity, and may further suffer in diagnostic accuracy [6]. Thus, considering the great advances made in diagnostic protein microarrays, we believe that there exists a major potential health care benefit if microarray technologies could be made amenable for portable, rapid point-of-care use.

A considerable hurdle for creating portable, easy to use planar microarray solutions appears to be the accommodation of many sequential assay steps on-chip, as well as the need for comprehensive sample mixing in order to avoid long assay times due to large diffusion coefficients of protein biomarkers. These challenges are largely avoided in immunochromatographic/lateral flow-based tests, and to this end we previously demonstrated the feasibility of combining a lateral flow/dipstick assay framework commonly used in simplex/low-plex rapid tests with protein microarray patterning for rapid and accurate determination of levels of bovine IgG raised towards mycoplasma infection [7] using a cell phone digital camera or consumer table top scanner for array imaging.

In this paper, we expand the concept into a general high-plexed lateral flow protein microarray (LFM) strategy, with 384 protein fragments (approximately 80–100 aa) [8] immobilized in a 24 × 16 microarray pattern on nitrocellulose. The protein fragments are a subset of antigens used for the large-scale generation of monospecific antibodies within the Human Protein Atlas (HPA) [9] where the aim is to by 2015 generate a set of highly specific antibody binders for the complete human proteome. In the HPA, bioinformatically designed protein fragments are employed as immunogens, as well as ligands in the subsequent affinity purification and quality control/validation of the generated antibody reagents. Together with the obvious benefit of providing reagents for determining presence or levels of virtually any expressed human protein in tissues or body fluids, there is ongoing work exploring the potential of using the protein immunogens as affinity binders for detection of disease-specific antibody populations [10]. Thus, investigating alternative microarray assay formats employing protein immunogens as affinity binders could result in improved strategies for quality control of raised antibodies as well as in novel methods for diagnostic antigen microarray tests.

In this work, by employing a prototype lateral flow protein microarray device, we were able to accurately determine levels of 26 individual HPA antibodies, as well as up to 8 concurrent specific IgG analytes in approximately 10 min total assay time. We argue that: (a) the demonstrated concordance with an established glass/fluorescence microarray assay; (b) the simplicity of the assay procedure drawing from the benefits of common lateral flow/dipstick tests; (c) the highly multiplexed capacity of the assay; (d) the short assay time; and (e) the convenient read-out options, make the presented assay framework an interesting tool for rapid quality control of raised antibody reagents, and may further find use in translating the capabilities of protein microarrays to point of care/low resource settings.

## 2. Results and Discussion

**Figure 1:**
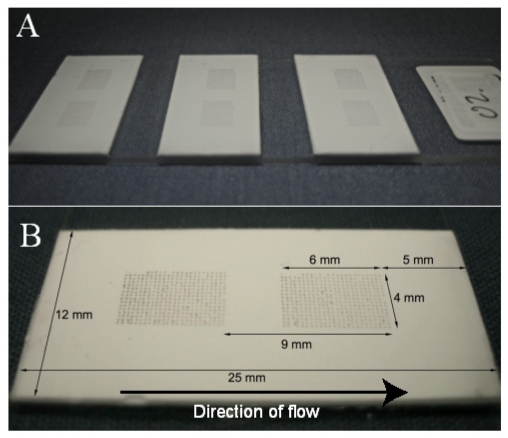
(**A**) Three cardboard-backed nitrocellulose strips attached to a glass array slide. Two arrays were printed per strip, but only the data from the upstream array was used in this study; (**B**) Here, an anti-HisABP antibody was used to reveal the pattern of the printed antigens which all had been recombinantly fused with a His_6_ABP-tag.

In this study, a novel protein antigen lateral flow microarray containing 384 different capture microspots patterned onto a nitrocellulose substrate and with a gold nanobead detection strategy (Figure 1) was evaluated in terms of absolute sensitivity and accuracy in comparison with a conventional glass/fluorescence-based microarray analysis of 26 IgG antibody species. We also investigated the method’s inherent variability as well as its ability to detect mixtures of up to eight different specific rabbit IgGs.

### 2.1. Analysis of 26 Specific Rabbit IgG and Assay Accuracy

A selection of 26 purified rabbit IgG isolates, diluted to 0.17–6.91 μg/mL was analyzed in triplicates on the lateral flow microarray as well as on the reference glass slide microarray (Table 1). It was found that both platforms could correctly identify all 26 antibodies individually, with signal to noise values of 12–181 (mean 100) for the LFM and 23–347 (mean 192) for the glass array. For five samples, an additional strong common signal was found by both platforms. Further, on five occasions the glass array detected an additional strong signal which was not found by the LFM, whereas the LFM only once picked up an additional signal which was not found by the glass array. This may suggest that the glass array is more sensitive to weaker interactions, which may or may not be beneficial depending on the application. While higher signal to noise values were recorded from the glass array, the assay procedure was easier to perform for the lateral flow array, and the difference in assay time was substantial, around 10 min for the lateral flow array compared to more than two hours for the glass array assay. Further, the distinct red color arising on the lateral flow array allowed convenient options of employing a table top scanner or cell-phone camera for the array imaging. We propose that the excellent concordance suggests that the sensitivity, accuracy and multiplexing ability of glass-based protein microarrays can be retained in the translation into immunochromatographic/lateral flow assay frameworks.

**Figure 2:**
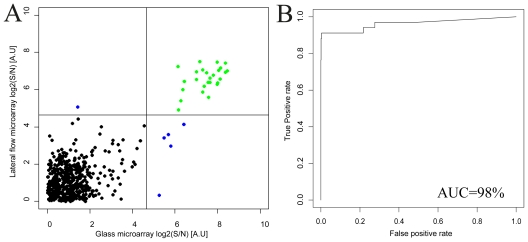
(**A**) Lateral flow and glass microarray results from the analysis of 26 antibody samples are compared. Green dots (in the top right quadrant) indicate concordant positive signals, black (bottom left quadrant) are concordant negative signals and blue dots (top left and bottom right quadrants) represent positive signals that only arose on one platform. All expected antibodies were detected by the correct antigen spot on both platforms. The Pearson correlation coefficient for the dataset was 0.74. Only signals higher than the mean for each array are shown; (**B**) A receiver-operator-characteristics analysis was performed, employing the vertical threshold for the glass array signals indicated in (**A**). The area under the curve (AUC) was found to be 98%, suggesting a very good binary classification concordance between the methods.

A good agreement between the lateral flow array and the glass array results was found (Figure 2) and the Pearson correlation coefficient was estimated to 0.74. Further, a receiver-operator-characteristics analysis was performed to reveal the concordance in terms of sensitivity and specificity for the comparison between the methods. While both the lateral flow and the glass array assays required thresholds of around 22 to accurately separate positive signals from negative, it was found that the area under the curve (AUC) could be estimated to 98%, indicating that the antigen microarray could be transferred from the conventional glass assay into the immunochromatographic/lateral flow platform with retained accuracy.

A moderate correlation coefficient is to be expected due to differences in substrate coupling to the protein antigens, as well as substantial differences in assay dynamics. However, the sensitive and specific binding of the sample antibodies on the corresponding antigen microspots should be retained, and this is indeed what the ROC analysis demonstrates.

### 2.2. Assay Sensitivity

**Figure 3:**
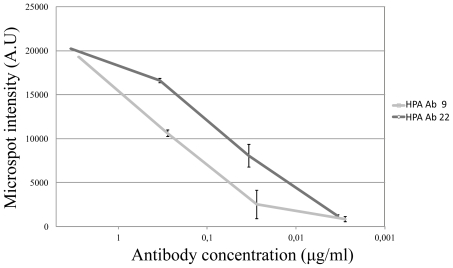
Dilution series of two antibodies, HPA Ab 9 and HPA Ab 22. The antibodies were diluted 1:100, 1:1000, 1:10.000 and 1:100.000 and the signal from the expected positive antigen spot was measured. It was found that a linear relation between the intensity and the log of the concentration was found between dilutions 1:100 and 1:10.000. As no significant signals were found at dilutions 1:100.000, it is suggested that the sensitivity may reside at or lower than around 30 ng/mL for the lateral flow analysis of the two antibodies.

Two antibodies (HPA Ab 9 and HPA Ab 22, due to suitably high initial concentrations) were selected for a sensitivity analysis. Each antibody was diluted 1:100, 1:1000, 1:10.000, 1:100.000 in assay buffer, and three replicates per antibody were run for each dilution. As compared with the negative control, it was found that the sensitivity could be estimated to around 30 ng/mL or better and that the response tended to be log-linear (Figure 3).

### 2.3. Assay Variability Investigation

From the triplicate runs of the total cohort of 26 specific IgG species included in this study (and under the assumption that the uncontrolled variation was Gaussian distributed), we calculated the standard deviation of each analyzed antibody. We found that the average coefficient of variation (%CV) was 13% [5%–21%]. The level of variability may impede highly resolved quantitative analysis, but could still be acceptable for semi-quantitative approaches.

### 2.4. Analysis of IgG Mixed Samples

**Figure 4:**
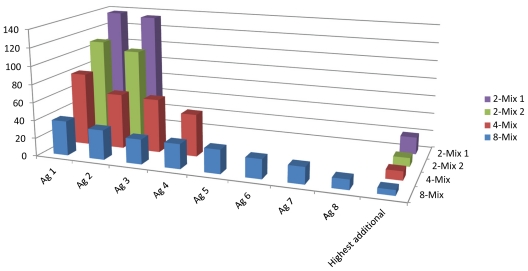
Four antibody mixes were prepared, two containing two antibodies, one containing four antibodies, and one containing eight antibodies. In all cases, the signal/noise on the expected antigen (Ag) spots were distinctly higher than the highest unexpected antigen signal on the same array, although the S/N decreased with increased number of antibodies in the mix.

In order to demonstrate the array’s capability of detecting an increasing number of antibody analytes, we created mixtures of 2, 2, 4 and 8 antibodies to be analyzed on the lateral flow microarray. It was found that the array could accurately identify all the constituents of the mixes, although the positive signal to noise ratios gradually decreased (Figure 4) due to increasing background/array mean intensities. The increased array mean was expected, and did conceivably arise due to the increased concentration of total IgG in the mixed samples, which amounted to around 15 μg/mL in total for the 8-mix, in comparison to between 0.17 and 6.9 for the individual antibodies (in the case of the antibody HPA Ab 26 which was analyzed at 6.9 μg/mL, the background was already substantial). Thus, it appears likely that at least 8 different antibody species may be detected simultaneously by the lateral flow microarray, given that the sample dilution keeps the total IgG concentration at or below a critical level (15 μg/mL in this study).

## 3. Experimental Section

### 3.1. Protein Antigens and Antibodies

All protein antigens were selected from the antibody quality control division of the HPA project [11]. The antigens are 80 to 100 amino acid long protein epitope signature tags that are computationally chosen from the protein encoding genetic sequence and fused with a His_6_-albumin binding protein domain [8]. Antigens are designed to exhibit low signal similarity to other human proteins and encode for no transmembrane regions. Following design, the antigens are recombinantly expressed in *E. coli* and the protein product subsequently affinity purified under denaturing conditions before being immunized into rabbits to stimulate antibody production. The antibodies are harvested from the rabbit after 4 months and purified by means of affinity chromatography using the immunogens/antigens as affinity ligands [12].

### 3.2. Buffers

Protein antigens were diluted in printing buffer (50 mM sodium carbonate-bicarbonate buffer + 49% glycerol, pH 7.4) before patterning on substrates. The assay buffer used for most dilutions and washes contained phosphate buffered saline (PBS) + 0.5% Tween20, together with 3% bovine serum albumin (Sigma) and 1% sucrose (Sigma) at pH 7.4.

### 3.3. Lateral Flow Microarray Substrate and Patterning

Cardboard-backed nitrocellulose membranes (HighFlowPlus90, Millipore) were cut into 12 by 25 mm strips and glued to 0.8 mm thick arraying slides (Arrayit) with off-the-shelf super glue (Loctite Super Glue Precision, Henkel). 384 individual protein antigen capture probes were then spotted onto the membranes using an Arrayjet Marathon (Arrayjet Ltd.) at 80 μg/mL in printing buffer. The array blocks were printed in a 16 by 24 spot layout with 280 μm distance between spot centers (Figure 1). Approximately 100 pL sample was deposited on each spot. The printed arrays could be stored dry at room temperature for up three months without apparent loss of sensitivity (data not shown).

### 3.4. Glass Microarray Patterning and Assay Procedure and Detection

The patterning of glass microarrays was carried out using the same printing protocol as for the lateral flow microarray, but using epoxy-derivatized glass slides (OPEpoxy slides, Captital Bio) as substrates. After printing, slides were allowed to rest at 37 °C for 24 h, after which slides were blocked in PBS + 0.1% Tween20 + 3% BSA for 1 h on a shaker at 160 rpm. Slides were then washed three times with PBS for 5 min each, followed by brief rinsing in deionized water and finally drying by spinning 2 × 3 min at 700 rpm. The assay procedure for the use of glass slide antigen arrays in the analysis and quality control of rabbit sera has been described elsewhere [12]. Briefly, slides were incubated with the antibody sample for 60 min on a shaker table at 150 rpm. An adhesive silicone mask (Schleicher and Schuell) was clamped on the slide in order to separate the 16 array blocks. Subsequently, the arrays were washed twice for 5 min on a shaker at 110 rpm with PBS + 0.1% Tween. Next, the arrays were incubated with a fluorescent secondary antibody (Goat anti-rabbit Alexa 647, Invitrogen) for 1 h at 4 ng/mL, followed by washing of the arrays for 5 min on a shake at 110 rpm with PBS + 0.1% Tween20. After the slide had been dried by means of spinning, it was scanned using an array scanner G25O5B (Agilent Technologies) and analyzed by means of the software GenePix Pro 5.1 (Axon Laboratories).

### 3.5. Lateral Flow Microarray Assay Procedure

A 1 mm thick line of grease (Spezialfett #3500, Heraeus) was applied 2 mm from the top end of each strip across the width of the membrane, forming the lower boundary of a sample drop-in area. The resulting hydrophobic barrier forced the sample to travel only through and not on top of the nitrocellulose membrane. A cotton sheet (Whatman) of around 1 × 2 cm was placed at the end of the membrane to serve as a fluid sink. Initially, the membrane was presoaked with 30 μL assay buffer in order to avoid non-specific binding and provide a homogeneous flow profile. Subsequently, 30 μL antibody sample was applied, followed by a 15 μL wash with assay buffer. Next, 30 μL of biotin-conjugated goat anti-rabbit F(ab′)2 (Jackson Immunoresearch) was applied, again followed by a 15 μL wash step. Finally, 30 μL of OD10 40 nm monoclonal goat anti-biotin coated gold particles (British Biocell International) diluted 1:3 in assay buffer was applied, followed by a 30 μL wash step. Each applied liquid step needed around 90 seconds to complete, giving a total assay time of around 10 minutes. As a quality control step to ensure all antigen spots had been printed satisfactorily, a 100 μg/mL rabbit anti-HisABP antibody (generated in house from HPA project) was applied to one strip instead of rabbit IgG solution, which resulted in binding to the His_6_ABP-tag present on all antigens (Figure 1). In all assays, each consecutive step was initiated immediately after the depletion of the previous step’s entire droplet into the drop-in area.

### 3.6. Image Acquisition and Data Analysis

After drying (1–5 min depending on the surrounding temperature and humidity), the arrays were scanned using a standard tabletop HP Scanjet 8270 (Hewlett Packard) at 1200 dpi. 16 bit gray scale tiff images were acquired by VueScan 8.6.34 (Hamrick Software) and subsequently inverted and analyzed using GenePix Pro 5.1 (Axon Instruments). Data analysis was performed with the statistical language R version 2.12 [13]. The median spot intensity subtracted by the inter-spot background was used for both lateral flow and glass microarray data. Signal to noise values were calculated by dividing each array microspot intensity with the array mean of background-subtracted spot intensities. For receiver-operator-characteristics analysis, a glass array threshold was selected that most accurately separated the expected (from the immunization pattern) positive signals from the expected negative ones, creating a binary vector of all antibody samples in the cohort. Subsequently, the corresponding array of sample data resulting from the lateral flow array analysis was compared to the binary vector employing the ROCR package in R.

## 4. Conclusions

Efforts to discover novel clinically useful biomarkers is continuously generating better molecular tools that through earlier and more accurate diagnosis and better disease monitoring may eventually greatly reduce the global burden of disease [14]. A key challenge lies in translating the wealth of improved diagnostic potential from research into clinical practice. While current microarray technologies are well suited to handle a high number of novel affinity binders, point of care tests are lagging behind in terms of multiplexing ability, sensitivity and diagnostic accuracy. Here, we present a novel device which draws its speed, portability and ease of use from the widely used lateral flow rapid test and the sensitivity, accuracy and multiplexing ability from the planar microarray. The sensitivity was found to be at or better than around 30 ng/mL, a level which should be acceptable in many IgG-based assays where disease-specific IgG are typically in the order of μg/mL or higher. The average variability was estimated to 13% CV which could be suitable at least for semi-quantitative analyses. Further, in comparison with a conventional glass/fluorescence microarray assay, we show that the concordance between the methods for a set of 26 rabbit IgG antibodies was excellent, with an AUC of 98%, suggesting that the lateral flow microarray could prove useful as an alternative in quality control for high-throughput antibody generation as well as for future potential rapid and portable diagnostic protein antigen microarrays. A brief qualitative comparison between the lateral flow array and glass array assay systems with regards to some relevant performance parameters can be found in table 2. We believe an important novel finding is that the nitrocellulose substrate, which has proven immensely useful for rapid and simple immunochromatography tests, appears amenable for high-density protein microarray analysis with retained diagnostic accuracy. Coupled to a detection strategy involving gold nanobeads, the LFM allowed for a flexible and convenient detection and read-out strategy along with the powerful analytic ability of the microarray. In future work we will select a similarly sized set of protein antigens which have demonstrated a clinically relevant diagnostic utility and evaluate the lateral flow microarray’s ability on clinical samples. We will also attempt to develop the device further for less manual operation and towards integration of filtration steps for whole-blood analysis and automated image analysis.

## Figures and Tables

**Table 1:** 26 Antibodies were analyzed with the lateral flow and glass microarray. For all antibodies, both methods correctly detected the IgG analyte with the expected antigen spot, although the signal to noise was usually greater for glass than LFM. In five cases, one additional antigen spot gave rise to a significant signal on both platforms (LG). Five times, the glass array gave rise to an additional antigen signal while no signal was seen on the LFM (G), whereas the reverse case only occurred once (L).

Antibody sample	Original concentration [μg/mL]	(S/N) LFM	(S/N) Glass	Additional signals	Comment
HPA Ab1	17	79	251	LG	
HPA Ab2	66	12	51		
HPA Ab3	80	18	85		
HPA Ab4	108	45	145		
HPA Ab5	113	110	221	LG	
HPA Ab6	116	59	156		
HPA Ab7	144	88	253	LG,G	
HPA Ab8	148	173	326		
HPA Ab9	340	123	325	G	
HPA Ab10	141	153	70		
HPA Ab11	104	181	142		High BG glass array
HPA Ab12	97	84	198		
HPA Ab13	94	94	128		High BG glass array
HPA Ab14	88	134	156	G	
HPA Ab15	85	125	128		
HPA Ab16	58	48	190		High BG glass array
HPA Ab17	100	17	23		Low signals on both platforms
HPA Ab18	93	143	278	G	
HPA Ab19	76	73	164		High BG glass array
HPA Ab20	50	120	177	G	High BG glass array
HPA Ab21	48	99	199	LG	High BG glass array
HPA Ab22	278	179	249	L	
HPA Ab23	164	95	277		
HPA Ab24	183	85	186		
HPA Ab25	188	131	261	LG	
HPA Ab26	691	130	347		High BG glass array
LG	Additional antigen spot positive for both methods				
G	Additional antigen spot positive for only glass array				
L	Additional antigen spot positive for only lateral flow array				

**Table 2:** Qualitative comparison of some aspects of the lateral flow and glass array assay formats.

	Glass array	Lateral flow array
Multiplexing ability	High	High
Sensitivity	High	Medium
Reproducibility	Medium	Medium
Cost/assay	Medium/High	Low
Assay time	~2 h	~10 minutes
Procedure difficulty	Medium	Low
POC potential	Low	High
